# Pain catastrophizing moderates the relationship between chronic pain and insomnia severity in persons with opioid use disorder

**DOI:** 10.3389/frsle.2023.1111669

**Published:** 2023-06-22

**Authors:** Melanie A. Baime, Prem Umang Satyavolu, Andrew S. Huhn, Jennifer D. Ellis

**Affiliations:** Department of Psychiatry and Behavioral Sciences, Johns Hopkins University School of Medicine, Baltimore, MD, United States

**Keywords:** opioid use disorder (OUD), insomnia, chronic pain, pain catastrophizing, moderation

## Abstract

**Study objectives:**

Chronic pain and insomnia commonly co-occur among individuals with opioid use disorder (OUD) and are associated with adverse treatment outcomes and reduced quality of life. Exploring factors that influence these relationships may help identify relevant treatment targets. The present study investigated whether pain catastrophizing moderates the presence of chronic pain and insomnia severity in individuals with OUD.

**Methods:**

Participants with OUD symptoms (*N* = 154) were recruited from Amazon's Mechanical Turk, and completed screening measures for chronic pain, insomnia, and pain catastrophizing. Moderation analyses were used to explore whether pain catastrophizing moderated the relationship between chronic pain and insomnia severity.

**Results:**

Results suggested that chronic pain was only associated with insomnia severity symptoms among individuals with higher levels of pain catastrophizing but was unrelated at lower levels of pain catastrophizing.

**Conclusions:**

These results suggest that pain catastrophizing may represent a modifiable risk factor among individuals with co-occurring OUD, insomnia, and chronic pain. Future longitudinal and experimental research that examines changes in insomnia, pain severity, and pain catastrophizing over time in OUD may be beneficial.

## Introduction

In 2020, ~2.7 million people in the United States had opioid use disorder (OUD) (National Institute on Drug Abuse, 2022), and the number of overdose deaths involving opioids has increased exponentially over the last two decades (U. S. Department of Health and Human Services, 2022). Individuals in treatment for OUD can benefit from efficacious treatments such as buprenorphine, methadone, or naltrexone (Blanco and Volkow, 2019); however, persistent mental and physical health symptoms can interfere with successful treatment. Sleep disturbances, including clinical insomnia, have emerged as a potential therapeutic target to improve OUD treatment outcomes (Huhn and Finan, 2021).

Insomnia is a common co-occurring condition in OUD, as well as a known side effect of opioid withdrawal (Huhn et al., 2022; Ellis et al., 2022a). In order to sleep, some individuals resort to using opioids to prevent this symptom of withdrawal. Agonist and partial agonist medications for opioid use disorder may ameliorate withdrawal-induced insomnia; however, insomnia and other sleep problems often persist even among patients receiving medication treatment for opioid use disorder (Wilkerson and McRae-Clark, 2021). Treating these sleep issues and understanding why and how they occur is imperative to preventing individuals from relapsing in order to avoid persistent sleep disturbances.

Chronic pain is a prevalent co-occurring condition in OUD and is associated with increased risk of sleep disturbances (Martinez et al., 2020). Additionally, chronic pain is often not addressed in opioid treatment programs, and many individuals with co-occurring chronic pain and OUD are more likely to report pain as a reason for OUD recurrence (Ellis et al., 2022b). As a result, chronic pain can continue to impact both sleep and opioid use, undermining otherwise effective treatments. Thus, identifying mechanisms by which chronic pain influences clinically relevant characteristics of OUD such as persistent sleep disturbances may improve outcomes.

Insomnia (Bei et al., 2016) and pain (Mun et al., 2019) are dynamic processes that interact in a bidirectional manner. For example, daily increases in sleep disruption may enhance pain (Moscou-Jackson et al., 2015), and attenuate analgesic effects of morphine (Smith et al., 2020), while increases in pain above one's typical level have been associated with greater disruptions in sleep continuity and greater subjective difficulties falling asleep. Thus, individuals experiencing both insomnia and clinically significant pain may experience symptoms that exacerbate one another and worsen over time. There is a need to better understand modifiable risk factors that may decouple the pain/insomnia relationship, in order to inform treatment and future longitudinal designs.

One potential moderator of the chronic pain/insomnia relationship is pain catastrophizing. Pain catastrophizing is defined as negative cognitive and emotional schema when an individual is experiencing or anticipating pain, and often involves feeling of helplessness and loss of control surrounding pain (Quartana et al., 2009). Previous research has identified a relationship between pain catastrophizing, daily self-reported pain, and opioid withdrawal in women who use opioids (Huhn et al., 2019), highlighting that pain catastrophizing may be a potentially modifiable risk factor (Schütze et al., 2018). Although pain catastrophizing has not previously been examined as a potential moderator of the chronic pain/insomnia symptoms relationship, previous work has tested interactions between pain intensity and opioid use on insomnia symptoms (Miller et al., 2018). Pain catastrophizing is a modifiable risk factor and can be targeted by existing psychological interventions (e.g., CBT for pain), making it an appealing target for future intervention research.

Insomnia and chronic pain are common comorbidities in persons with OUD. However, the conditions under which insomnia and chronic pain are more likely to co-occur in OUD remains a question in need of further study. The current study explores pain catastrophizing as a moderator between screening positive for chronic pain and insomnia severity in persons with OUD. It was expected that (1) individuals with chronic pain would exhibit increased insomnia severity, and (2) that this risk would be particularly high among individuals with heightened pain catastrophizing.

## Materials and methods

### Participants

Participants were adults living in the United States recruited from Amazon Mechanical Turk (AMT) between May to August of 2020. AMT is an online crowd-sourcing platform that has successfully been used to target hard-to- reach populations, including individuals with substance use disorders (Strickland and Stoops, 2019). All individuals who completed the survey endorsed being in treatment or recovery for OUD. To help ensure adequate individuals were recruited among this population, anyone with an approval rating less than 90% or not living in the United States were fielded out as ineligible to enroll. Participants completed an initial eligibility screener where eligibility questions were blinded by distractor questions (demographics, other health conditions, etc.). To be eligible, they needed to be over 18 years old and currently in treatment or recovery for OUD. For additional information, see Ellis et al. (2022a). The study was formally submitted to and acknowledged by the Johns Hopkins School of Medicine Institutional Review Board as exempt from human subjects research due to the deidentified nature of data collection.

### Measures

#### Demographic characteristics

Information regarding age, sex, race, marital status, and current household income was collected. Participants also responded to questions about lifetime use of various substances in addition to opioids.

#### Chronic pain screen

The 6-item Graded Chronic Pain Scale, Revised (GCPS-R) (Von Korff et al., 2019), was used to index the presence of chronic pain over the past 3 months. In the present study, the measure was dichotomized, and participants who screened negative were compared to participants who screened positive for grade 1 chronic pain or higher.

#### Insomnia screen

Using a 0–4-point Likert scale, symptoms of insomnia over the past 2 weeks were measured using the 7-item Insomnia Severity Index (ISI) (Bastien, 2001). The outcome was examined continuously.

#### Pain catastrophizing

The Pain Catastrophizing Scale (PCS) (Sullivan et al., 1995) is a thirteen item self-report measure for adults designed to assess catastrophic thinking related to pain. Each item uses a 0–4-point Likert Scale and the total score was used for analyses in this study.

### Data analysis

Data were screened for normality and outliers. Descriptive and bivariate correlation analyses were performed in SPSS, version 27. Next, moderation analyses were conducted using the PROCESS Macro, Version 4.0 (Hayes, 2022), Model 1. This moderation analysis explored pain catastrophizing as a potential moderator between screening positive for chronic pain and insomnia severity. For both moderations, the Johnson-Neyman formula was used to probe significant interactions. We conducted both unadjusted and adjusted analyses (controlling for age and sex). All analyses were considered significant at *p* < 0.05.

## Results

Demographic characteristics are presented in Table 1. In the current study, 60.4% of participants were male and 39.6% of participants were female. Regarding race, 70.8% of the participants identified as white/Caucasian, 12.3% identified as Black/African American, 4.5% identified as American Indian, 5.8% identified as Asian, 3.2% identified as Native Hawaiian/Pacific Islander, and 3.2% identified as more than once race. As for marital status, 36.4% of participants reported being single/widowed/separated/divorced and 63.6% reported being married/remarried. Yearly income was collected as well; 35.1% of participants reported making $30,000 or less, 35.7% reported making between $30,000 and $60,000 per year, 14.9% reported making between $60,000 and $90,000 a year, and 14.3% reported making above $90,000 per year. In bivariate analyses, individuals with greater insomnia symptoms reported greater pain catastrophizing (*r* = 0.73, *p* < 0.001), and were marginally more likely to screen positive for chronic pain [*t*_(152)_ = 1.91, *p* = 0.058]. Men and women did not differ with regard to insomnia severity, likelihood of screening positive for chronic pain, or pain catastrophizing. Age was not related to any variables of interest.

**Table 1:** Demographic characteristics.

**Variable**	**N(%) or M(SD)**
Sex (female)	61 (39.6%)
Age–M(SD)	35.13 (8.42)
Hispanic ethnicity	31 (20.1%)
**Race**	
White/Caucasian	109 (70.8%)
Black/African American	19 (12.3%)
American Indian	7 (4.5%)
Asian	9 (5.8%)
Native Hawaiian/Pacific Islander	5 (3.2%)
More than one race	5 (3.2%)
**Marital status**	
Married or remarried	98 (63.6%)
Never married	46 (29.9%)
Divorced/separated	9 (5.8%)
Widowed	1 (0.6%)
**Income**	
$0–$30,000	54 (35.1%)
$30,001–$60,000	55 (35.7%)
$60,001–$90,000	23 (14.9%)
$90,001+	22 (14.3%)
Employment (% employed full or part time)	135 (87.7%)
**Current medication for OUD treatment**	
None	55 (35.7%)
Methadone	38 (24.7%)
Buprenorphine/Suboxone	18 (11.7%)
Extended release buprenorphine	3 (1.9%)
Naltrexone	18 (11.7%)
Extended release Naltrexone/Vivitrol	22 (14.3%)
Insomnia severity–M(SD)	13.79 (6.12)
Proportion screening positive for chronic pain	38 (24.7%)
Pain catastrophizing–M(SD)	22.89 (13.28)

### Moderation analysis

**Figure 1:**
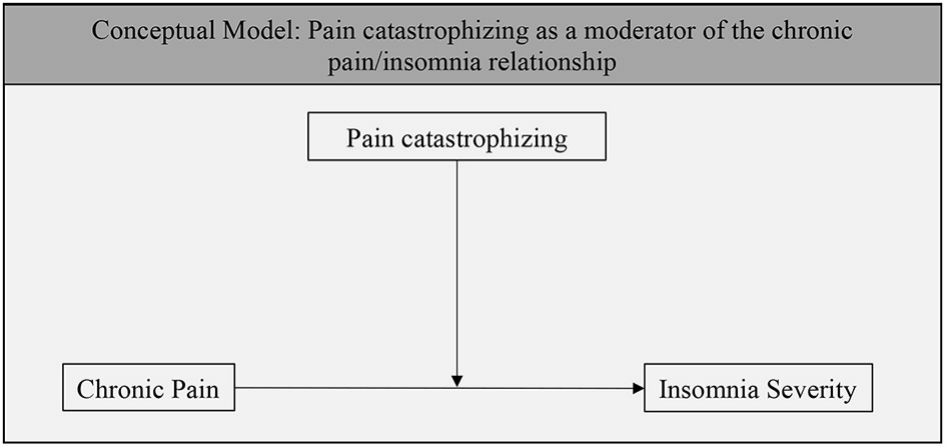
The above diagram shows the conceptual model guiding these analyses.

Moderation analyses are presented in Table 2, and the conceptual model is presented in Figure 1. A significant interaction was observed between screening positive for chronic pain and degree of pain catastrophizing *(B* = 0.15, *p* = 0.031), such that chronic pain was only related to insomnia severity among individuals with high levels of pain catastrophizing. Follow-up tests using the Johnson-Neyman formula indicated that the slope of chronic pain on insomnia severity was significant and positive at high levels of pain catastrophizing (i.e., over 38.2 on the pain catastrophizing scale), but was unrelated at lower levels of pain catastrophizing. This value represents over a standard deviation above average values of pain catastrophizing reported in previous work (e.g., Sullivan et al., 1995; M = 20.9, SD = 12.5) and over a standard deviation above the mean in the present study. The interaction effect remained significant when demographic characteristics (i.e., age and sex) were included in the model as covariates (*B* = 0.14, *p* = 0.042).

**Table 2:** Pain Catastrophizing as moderator of the chronic pain/insomnia relationship.

**Variable**	**Unadjusted models estimate (95% CI)**	**Adjusted models estimate (95% CI)**
Chronic pain (positive screen)	−0.01 (−1.62–1.61)	0.09 (−1.53–1.71)
Pain catastrophizing	0.30 (0.25–0.36)^**^	0.31 (0.26–0.37)^**^
Chronic pain x pain catastrophizing	0.15 (0.01–0.29)^*^	0.15 (0.01–0.28)^*^
Sex	-	0.06 (−0.07, 0.08)
Age	-	−1.12 (−2.49, 0.35)

^*^denotes a *p* < 0.05, ^**^denotes a *p* < 0.01.

## Discussion

Chronic pain and insomnia are both prevalent and highly comorbid among individuals with OUD. Identifying factors (e.g., pain catastrophizing) that may influence the relationship between these conditions can help inform treatment. The present study sought to test whether pain catastrophizing moderates the relationship between chronic pain symptoms and insomnia severity. Results suggested that chronic pain was only associated with insomnia symptoms among individuals with higher levels of pain catastrophizing symptoms. The PCS score of 38.2 in the current study represents a higher-than-average level of pain catastrophizing, even in relation to other individuals with chronic pain (Osman et al., 2000) These analyses highlight pain catastrophizing as a potentially relevant treatment target and area for future study in OUD.

The present study highlights the importance of assessing co-occurring symptoms, and providing integrated care to individuals in OUD treatment. Previous work suggests that nearly two-thirds of individuals with chronic pain in OUD treatment reported that their program did not assess for or treat their chronic pain, and that their pain influenced OUD recurrence (Ellis et al., 2021). Results from this study extend upon these findings by highlighting that individuals with chronic pain and OUD who experience high pain catastrophizing may also be more likely to experience co-occurring insomnia symptoms. It may be beneficial to provide additional training and educational opportunities for providers who work with individuals with co-occurring OUD and chronic pain. Given the high prevalence of chronic pain and insomnia, treatment centers may consider developing a plan for their facility to determine a process to identify and triage individuals entering treatment with OUD, chronic pain, and insomnia.

There are a number of treatment modalities that are efficacious in reducing pain symptoms, that should be explored further in populations with co-occurring OUD and chronic pain. For example, cognitive behavioral therapy (CBT) for pain involves identifying and altering maladaptive thoughts and beliefs, and utilizes various techniques to change the behavioral patterns and emotional responses to pain (Bryson et al., 2015). Of note, CBT-P has been effective in reducing the use of sleep medication in individuals who suffer from insomnia and fibromyalgia (McCrae et al., 2020), making this a promising intervention to extend to populations with chronic pain and OUD. Pain self-management skills, which help individuals develop and implement techniques such as pain scaling or the ability to recognize spikes in pain (Barry et al., 2019) may also be helpful. Interdisciplinary pain rehabilitation can also be useful to address pain catastrophizing, as this multi-modal approach has been shown to improve a number of outcomes including pain interference, pain catastrophizing, quality of life, depression and insomnia (Craner et al., 2020; Hobelmann and Huhn, 2021). Future work should explore the feasibility and acceptability of these interventions in populations with OUD, particularly with individuals who have multiple comorbidities that may be the focus of clinical attention.

Another relevant direction for future work could include examining the extent to which pain catastrophizing influences the dynamic relationship between pain and insomnia. For example, future work could leverage ecological momentary assessment (EMA) to collect daily measurements of pain and insomnia symptoms. This work could explore whether daily changes in pain catastrophizing influences daily association between pain intensity and insomnia. There has been increased attention in identifying individual differences in the variability and stability of pain symptoms (Mun et al., 2019). Future work could extend these findings to pain catastrophizing by exploring whether individuals with persistent and stable pain catastrophizing confer an elevated risk of co-occurring symptoms (e.g., insomnia) or ongoing opioid use.

Although the current study has provided insight into various aspects of chronic pain and insomnia, it should be considered in context of certain limitations. A cross-sectional sample was used in which participants' symptoms were self-reported. A longitudinal study would provide a platform for more comprehensive evaluation of insomnia and pain catastrophizing in the context of OUD. Another limitation is that the time frame in which the three main measures were completed by participants were different, and it is possible that this influenced the results. Actigraphy devices can be used as a non-invasive way to monitor sleep and physical activity in a person's everyday life outside of a laboratory. For insomnia, while self-report measures are essential, it is also important to assess clinical diagnoses and lab-based pain measures. Further, future studies should explore other moderators, such as mood. A convenience sample was used for OUD screening and the nature of this data collection precluded specific information on treatment settings and clinical diagnoses. Likewise, a physician-confirmed diagnosis of chronic pain was not provided and should be a key component in future studies. Additionally, the samples size was relatively small and may limit generalizability. Additionally, data surrounding culture and the impact it has on participant functioning will aide researchers in developing a program that will be generalizable to the larger OUD population while accounting for cultural and social factors specific to individuals.

In conclusion, this study is one of the first examinations of individuals with OUD that examines pain catastrophizing as a moderator between chronic pain and insomnia symptoms. Results suggest that individuals who screened positive for chronic pain and report high levels of pain catastrophizing are more likely to have more severe insomnia symptoms. Continuous assessment and culturally-sensitive individualized treatment for individuals with co-occurring chronic pain, insomnia, and OUD will allow for improved wellbeing as well as maintenance and reduction of symptoms for better overall health and functioning.

## Data availability statement

The raw data supporting the conclusions of this article will be made available by the authors, without undue reservation.

## Ethics statement

The study was formally submitted to and acknowledged by the Johns Hopkins School of Medicine Institutional Review Board as not human subjects research due to the deidentified nature of data collection.

## Author contributions

MB and PS: writing (original draft) and conceptualization. AH: writing (review and editing), supervision, conceptualization, and resources. JE: writing (review and editing), formal analysis, supervision, and conceptualization. All authors contributed to this manuscript and approved the final version.
